# Clinical evaluation of a fully electronic microfluidic white blood cell analyzer

**DOI:** 10.1371/journal.pone.0296344

**Published:** 2024-01-18

**Authors:** Jianye Sui, Zhongtian Lin, Shahriar Azizpour, Fei Chen, Sunanda Gaur, Kelly Keene, Farzad Soleimani, Tanaya Bhowmick, Zubaid Rafique, Mehdi Javanmard

**Affiliations:** 1 RizLab Health, Inc., Princeton, New Jersey, United States of America; 2 Department of Pediatrics, Rutgers Robert Wood Johnson Medical School, New Brunswick, New Jersey, United States of America; 3 Department of Emergency Medicine, Baylor College of Medicine, Houston, Texas, United States of America; 4 Department of Medicine, Rutgers Robert Wood Johnson Medical School, New Brunswick, New Jersey, United States of America; 5 Department of Electrical and Computer Engineering, Rutgers University, Piscataway, New Jersey, United States of America; The Ohio State University, UNITED STATES

## Abstract

The White Blood Cell (WBC) count is one of the key parameters signaling the health of the immune system. Abnormal WBC counts often signal a systemic insult to the body such as an underlying infection or an adverse side effect to medication. Typically, the blood collected is sent to a central lab for testing, and results come back within hours, which is often inconvenient and may delay time-sensitive diagnosis or treatment. Here, we present the CytoTracker, a fully electronic, microfluidic based instant WBC analyzer with the potential to be used at point-of-care. The CytoTracker is a lightweight, portable, affordable platform capable of quantifying WBCs within minutes using only 50 μl of blood (approximately one drop of blood). In this study, we clinically evaluated the accuracy and performance of CytoTracker in measuring WBC and granulocyte counts. A total of 210 adult patients were recruited in the study. We validated the CytoTracker against a standard benchtop analyzer (Horiba Point of Care Hematology Analyzer, ABX Micros 60). Linear dynamic ranges of 2.5 k/μl– 35 k/μl and 0.6 k/μl– 26 k/μl were achieved for total WBC count and granulocyte count with correlation coefficients of 0.97 and 0.98. In addition, we verified CytoTracker’s capability of identifying abnormal blood counts with above 90% sensitivity and specificity. The promising results of this clinical validation study demonstrate the potential for the use of the CytoTracker as a reliable and accurate point-of-care WBC analyzer.

## Introduction

The complete blood count (CBC) is the most routinely performed blood test in the clinical laboratory, requiring benchtop hematology analyzers and a full venous blood draw from the patient. Two of the most important subsets of parameters obtained in the CBC is the white blood cell (WBC) count and granulocyte count (predominantly consisting of the neutrophils), which is an important indicator of the state of health of the immune system [1–5]. WBC monitoring is important for numerous different indications in oncology [6, 7], infectious disease [4, 5, 8, 9], and mental health [10, 11]. For example, cancer patients on chemotherapy and various anti-cancer therapeutics often require frequent obtaining of their white blood cell and neutrophil (or granulocyte) counts since leukopenia and neutropenia are among the key side effects [7, 12, 13]. If they happen, antibiotic prophylaxis would be warranted. Another important indication where regular monitoring of neutrophil counts is necessary is for patients on psychiatric drugs (e.g. Clozapine for schizophrenia patients) [14]. To date, Clozapine is the most effective medication in treating schizophrenia, however, one of the major side-effects is neutropenia [10, 11, 15]. The FDA REMS (Risk Evaluation and Mitigation Strategies) program applies to all clozapine medications available on the market and a centralized system is in place to monitor patients and prevent/manage clozapine-induced neutropenia [16]. Administrative difficulties (including challenges of frequent laboratory testing), however, can cause people to miss doses, thus putting patients at risk for relapse, rehospitalization, and other potentially devastating outcomes [17].

White blood cell monitoring is also particularly important in antibiotic stewardship, which promotes the judicious use of antibiotics to prevent antimicrobial resistance [18, 19]. The rise of antimicrobial resistance (AMR) has emerged as one of the leading causes of death worldwide [20]. It is anticipated that by 2050, 10 million people will die per year of AMR [21]. Antimicrobial resistance is growing, while antimicrobial drug development is slowing [22, 23]. Now more than ever, antibiotic stewardship is of utmost necessity to prevent antimicrobial resistance, while improving patient outcomes. The key driver of AMR is the overuse of antibiotics [24, 25]. The use of antibiotics prompts the selection process that the bacteria with antimicrobial resistance can survive and even multiply [26]. According to the Centers for Disease Control and Prevention (CDC), between 30% to 50% of antibiotic usage is either unnecessary or inappropriate [27]. One of the key culprits of antibiotic overuse, particularly in the outpatient (or office) setting, is the use of antibiotics to treat viral infections that have been mistakenly diagnosed as bacterial infections [20, 24, 26]. Rapid monitoring of white blood cell counts can serve as an aid to clinical professionals in proper prescription of antibiotics [28, 29]. WBCs or leukocytes are critical parts of the immune system to protect the body against infections and other diseases. WBC counts fluctuate in the immune response to fight infections. It has been widely shown in the literature that high white blood counts, high neutrophil percentages, high neutrophil-to-lymphocyte ratios, and relatively low lymphocyte percentages tend to correlate with bacterial infections [30–35]. As an example, Lavoignet *et al*. conducted a retrospective and observational study to explore using the WBC count with differentials in diagnosing bacterial infections in Emergency Department (ED) [36]. They demonstrated that neutrophils and total WBC count were the two most useful leukocyte parameters for the diagnosis in the ED. In addition, the neutrophil-to-lymphocyte count ratio (NLCR) has been investigated as an indicator for the diagnosis of bacterial infections in many studies [32–34, 37]. A retrospective study showed that NLCR could be a tool in diagnosing bacterial infection among hospitalized patients with fever [35]. Evidence in the literature also shows that white blood counts with differentials can serve to assess infectious disease severity [8, 9, 38]. A high WBC count is also one of most important metrics in the SIRS criteria and can indicate the onset of sepsis [39, 40].

Challenges faced by medical practitioners and patients include the large volume of blood that must be obtained through a needle stick by a phlebotomist, the time required to transport the specimen to a central lab to perform analysis and make the results available for the medical practitioner. Miniaturization of blood cell analysis can enable testing in settings such as professional point of care areas, patient bedsides, physicians’ offices, and ultimately even home use, allowing for instant results for both the patient and physician. Shortening the feedback time between patient sample collection and results can have many advantages for both patients and medical practitioners, such as improved clozapine adherence for schizophrenia patients, rapid assessment of infectious disease severity, and stratification of patients at risk for severe infections, among many possibilities.

**Fig 1:**
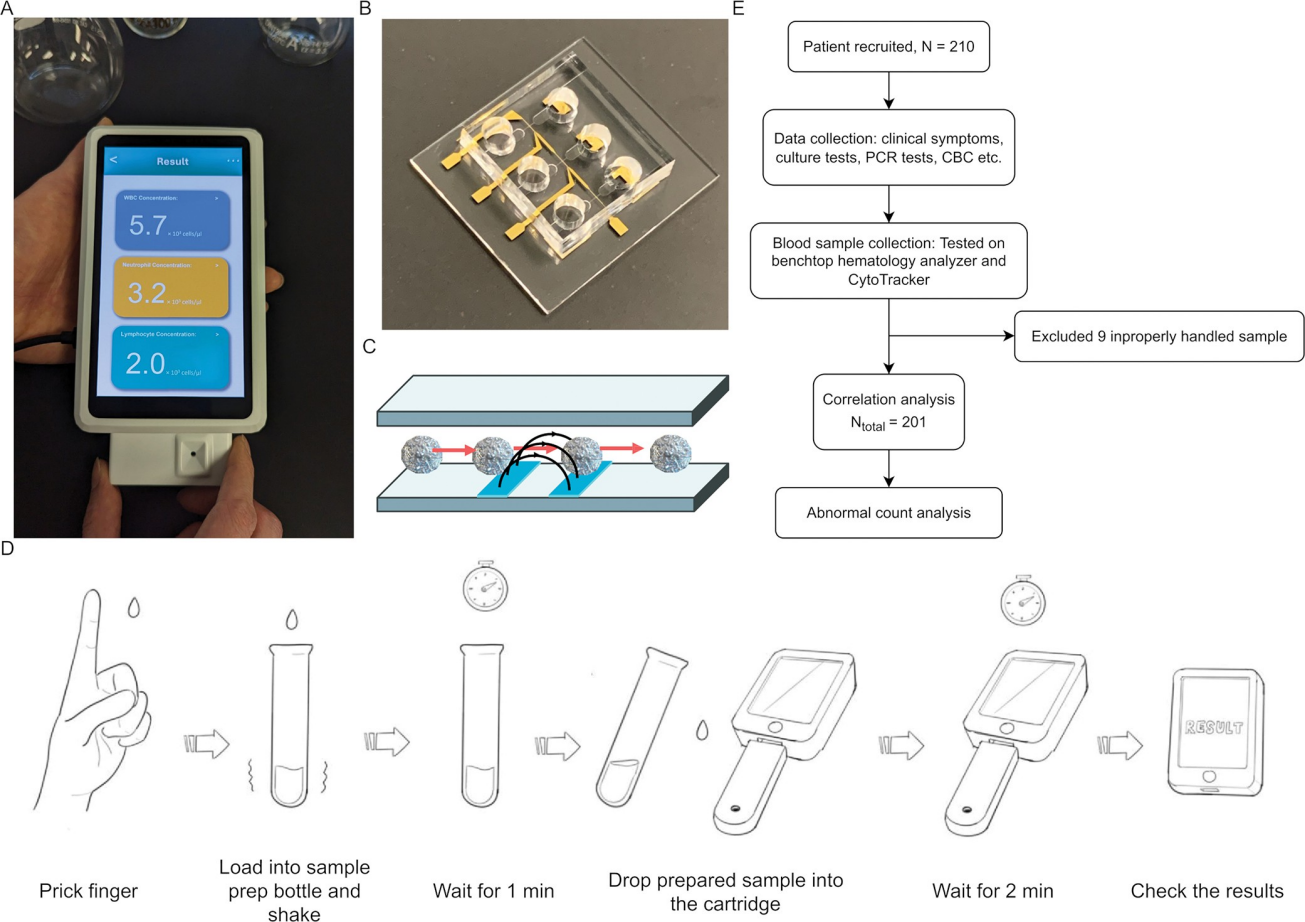
Study overview. (A) Image of CytoTracker device prototype. (B) An image of the CytoTracker microfluidic impedance cytometer. (C) Schematic of sensing mechanism. (D) A diagram of the proposed user workflow. Drop of blood is obtained from patient, then placed into sample processing tube for lysis of red blood cells. After waiting for one minute, several drops of processed blood are squeezed into the test strip (plugged into device). After waiting for two minutes, the result is available for the user. (E) An overview of study workflow. 210 adult patients with symptoms were recruited. Nine patients were excluded due to the sample being improperly handled during shipment. As a result, the final analysis was obtained from 201 patients.

The CytoTracker is a fully electronic miniaturized platform consisting of a reusable readout device and a disposable test strip (Fig 1A). This CytoTracker consists of a microfluidic impedance cytometer (MIC) test strip (Fig 1B) that works in conjunction with a miniaturized lock-in-amplifier readout circuit to detect WBCs, granulocytes (predominantly neutrophils), and also lymphocyte levels. We have described details of the microfluidic impedance cytometry system in prior publications, where we demonstrated implementation of the platform both in portable(handheld) [41] and wearable form factor [42]. Here we focus on clinical validation of the platform. Fig 1A shows the current model of the prototype. The clinical study presented here was performed using an older model of the device. The system uses only 50 μl of the blood sample, and the results are obtained in less than five minutes. To assess the performance of CytoTracker on patient blood samples, we conducted a study in collaboration with three clinical sites (Baylor College of Medicine, Rutgers Robert Wood Johnson Medical School, and BioIVT, Inc). In this study, given the need to ship samples from the clinical sites to RizLab Health for analysis, we benchmarked test strip performance using venous blood. Capillary blood needs to be analyzed within hours after collection, otherwise cell count measurements show wide variation over time, which was not feasible at the time given the study setup. We evaluated the reliability and accuracy of the CytoTracker in measuring WBC and granulocyte counts in patients with confirmed viral and bacterial infections; and investigated the CytoTracker’s accuracy in flagging abnormal blood counts. Additionally, we assessed the differences in cell counts between inpatients and outpatients, thus showing that the CytoTracker can potentially be used for infectious disease severity assessment as well.

## Materials and methods

### Study design and settings

We obtained whole venous blood samples from patients from three different clinical sites: Baylor College of Medicine (n = 53), Robert Wood Johnson Medical School (n = 99) and BioIVT (n = 58). BioIVT is a biological product provider. The samples obtained by BioIVT, inc. were from outpatients from a network of clinical sites. The samples obtained from BCM were from emergency room patients (recruited under an IRB for an observational study). The samples obtained from Rutgers RWJ were from emergency room and ICU patients (discarded samples obtained under a retrospective study). Venous blood samples were collected from May 2021 to November 2021. The study protocol was approved by Institutional Review Board for Baylor College of Medicine and Affiliated Hospitals (H-49795; ClinicalTrials.gov NCT05090319), Rutgers University electronic Institutional Review Board (Pro2021001264), and Western Institutional Review Board (WIRB® Protocol #20170439). Written consent was obtained by patients recruited by Baylor and BioIVT. The study at Rutgers was retrospective, and thus patient consent was not required.

All patients enrolled in the study were adults (>18 years) with suspected/confirmed viral (COVID-positive or other viral infection) and bacterial infections (lower UTI, pneumonia, septicemia, appendicitis, and other relevant bacterial infections), confirmed per standard of care (PCR test for viral, culture test for bacterial, or clinical presentation). Excluded from the study were patients with known white blood cell, neutrophil, and lymphocyte disorders, also active cancer patients and patients who received chemotherapy for solid tumors in the last three months, and pregnant patients.

Venous blood samples were collected from patients for analysis using both the CytoTracker and a Horiba ABX Micros 60 Hematology Analyzer (FDA-cleared device). The sample was stored at 4°C before overnight shipping. The sample tubes were wrapped with an absorbent pad and bubble wrap and placed in a Styrofoam box with cold packs during the shipment. All samples were analyzed at RizLab health within 72 hours after collection from patients. Deidentified information was abstracted from the medical chart including CBC values (assessed by the clinical laboratory), diagnostic lab tests, body temperature, date of sample collection, drug treatments (duration, dose), diagnosis for admission, and clinical status.

A sample size of greater than 150 patients was determined to achieve a high statistical power of greater than 99%. This sample size was selected to detect a significant correlation between the measurements obtained from the two devices. With this large sample size, the study’s ability to provide robust evidence regarding the relationship between cell counts is enhanced, supporting the statistical generalizability of the study.

### CytoTracker test and data analysis

Samples were tested using CytoTracker microfluidic test strips (Rizlab Health, Princeton, NJ). First, a 50 μl aliquot of EDTA-anticoagulated whole blood sample was processed using the CytoTracker sample preparation kit which involves lysing the red blood cells and quenching the reaction to prevent WBC lysis. Red blood cells were lysed, and the debris was reduced in preparation. Next, a 60 μl aliquot of the product was transferred to the microfluidic cartridge. The cartridge was connected to a read-out device for data acquisition and initial processing. Sample measurement from the microfluidic impedance cytometry chip took five minutes. WBC and granulocyte counts were obtained and benchmarked. Passing-Bablok regression was performed to model the relationship between the results acquired by the Horiba ABX micros 60 hematology analyzer and the CytoTracker, and the correlation coefficient was calculated. An α level of 0.05 was used for all analyses. Due to the non-normal distribution, the Mann-Whitney U test was performed to evaluate the difference in total WBC concentration and granulocyte concentration between inpatient and outpatient blood samples.

## Results

### Patient characteristics

Samples were obtained from 210 patients with bacterial or viral infections (Fig 1). Nine patients were excluded due to the sample being improperly handled during shipping. CytoTracker performance benchmarking was performed on 201 patient samples, 51 from BioIVT, 53 from Baylor College of Medicine, and 97 from Robert Wood Johnson Medical School (Table 1). The baseline characteristics of all patients from the three sites are summarized in Table 1. The median age was 54 (Q1-Q3 40–68), with 106 males (53.5%) and 82 females (46.5%). Of the total, 44.4% were white, 17.7% were African American, and 37.9% were patients of other races.

**Table 1:** Baseline characteristics.

		Overall	BioIVT (Outpatient)	Baylor (Inpatient)	RWJ (Inpatient)
		n = 201	n = 51	n = 53	n = 97
Type of Study			Prospective	Prospective	Retrospective
Age, median (Q1-Q3)		54 (40.3–67.8)	53 (41.8–68.8)	45 (36.3–55.0)	61 (46.0–71.0)
Gender (Male), N (%)		106 (52.7)	20 (39.2)	23 (43.4)	63 (64.9)
Race, N (%)	White	88 (43.8)	36 (70.6)	1 (1.9)	51 (52.6)
	African American	35 (17.4)	1 (2.0)	19 (35.8)	15 (15.5)
	Other	78 (38.8)	14 (27.5)	32 (60.4)	31 (32.0)
Infection type, N (%)	Viral	110 (54.7)	17 (33.3)	47 (88.7)	46 (47.4)
	Bacterial	91 (45.3)	34 (66.7)	6 (11.3)	51 (52.6)
WBC (10^3^ cells/μL), median(Q1-Q3)		7.5 (5.2–11.1)	6.3 (5.4–8.4)	6.7 (3.3–9.0)	9.2 (6.0–14.8)
Granulocyte (%), median(Q1-Q3)		67.2 (56.5–78.0)	57.7 (49.0–66.4)	66.3 (56.2–75.8)	71.1 (62.2–80.2)
Lymphocyte (%), median(Q1-Q3)		22.2 (14.6–31.0)	30.2 (26.5–37.8)	24.6 (16.5–33.8)	16.8 (11.8–23.8)
Monocyte (%), median(Q1-Q3)		8.7 (5.3–13.8)	9.2 (6.4–14.8)	7.8 (4.1–12.5)	9.3 (5.5–14.1)

### Precision, bias, and linearity

**Fig 2:**
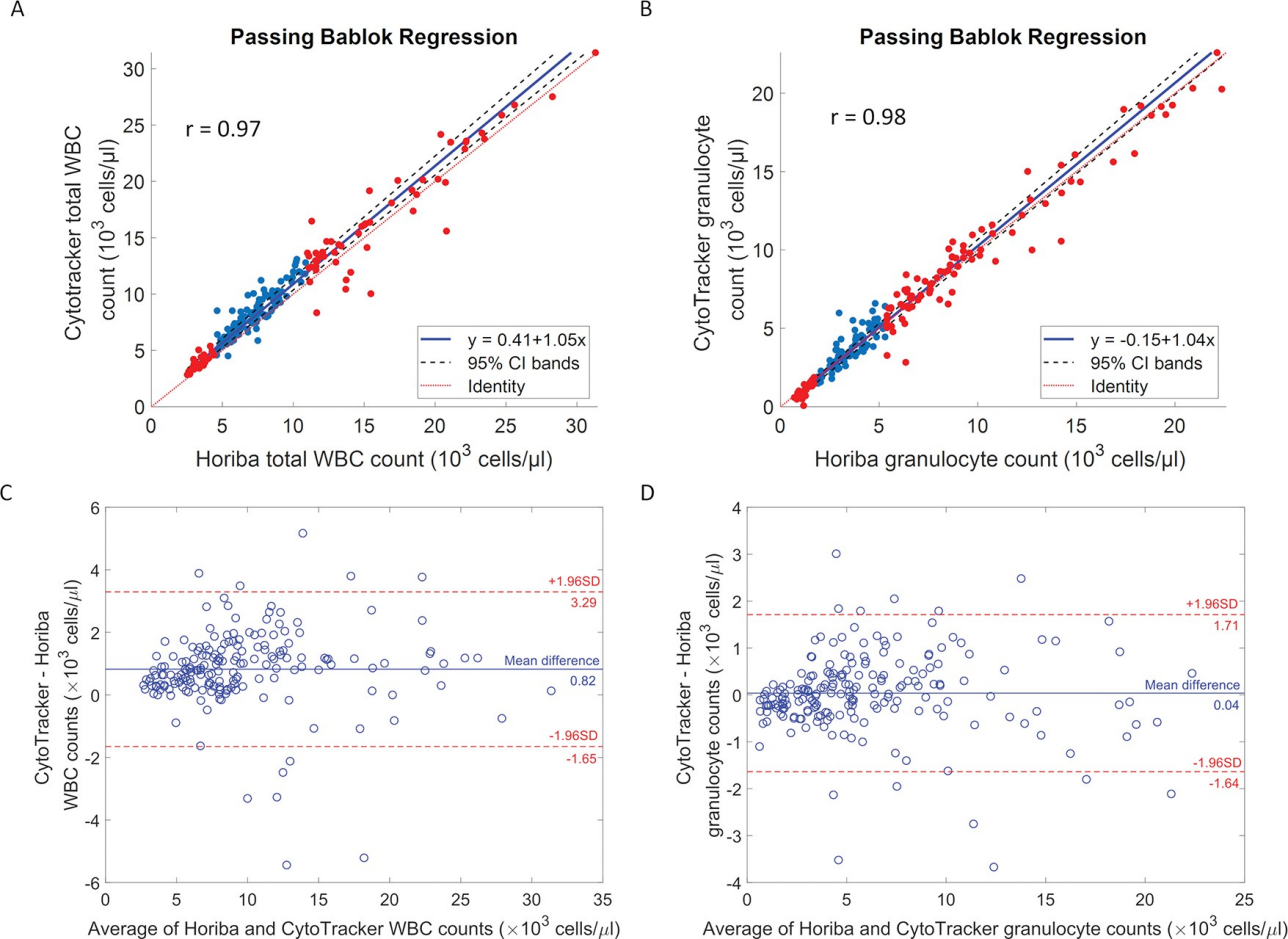
Passing-Bablok regression analyses and Bland-Altman plots. Passing-Bablok regression analyses between Horiba results and CytoTracker results. (A) Total WBC count: slope: 1.05 (95% CI 1.02 to 1.08) intercept: 0.41 (95% CI 0.15 to 0.65) (B) granulocyte count: slope: 1.04 (95% CI 1.01 to 1.07) intercept: -0.15 (95% CI -0.30 to -0.04). The x-axis is the measured cell counts obtained using the Horiba hematology analyzer, while the y-axis is the measured cell counts obtained using the CytoTracker. The red dots represent samples flagged for abnormal counts outside of the normal range for WBC (4.5 k/μl—11 k/μl) and granulocytes (1.2 k/μl—6.8 k/μl). The solid blue line represents the regression line, the dashed red line represents the line of identity, and 95% confidence intervals are represented by the black dashed lines. Bland-Altman plots present level of agreement between CytoTracker and Horiba on WBC count (C) and granulocyte count (D), with the mean of the differences (blue solid line) and ± 1.96 SD limits (red dashed lines).

The aim of this work is to clinically validate that the CytoTracker cartridges can accurately measure the WBC count and granulocyte count, for assessing if cell levels in patients are healthy or abnormal. We benchmarked CytoTracker performance against an FDA-cleared reference hematology analyzer and focused primarily on three metrics: linearity, precision, and accuracy in flagging abnormal cell levels. We tested all patients (n = 201) with Horiba ABX micros 60 hematology analyzer to obtain the true value before measuring blood samples with CytoTracker. A 50 μl aliquot of blood sample was lysed, then pipetted into the CytoTracker cartridge. After 5 minutes of measurement, the results were calculated based on captured signals. We performed Passing-Bablok regression analysis and correlation analysis on the results of total WBC count and granulocyte count between the two devices. Passing-Bablok regression is a nonparametric regression analysis suitable for method comparison studies. The assumption of the linear relationship between the two devices’ results was validated using the modified cumulative sum (CUSUM) test described by Passing and Bablok [43]. The measured CytoTracker dynamic range for WBCs was 2.5 k/μl—35 k/μl and for granulocytes was 0.6 k/μl—26 k/μl. The results are shown in Fig 2A and 2B. The x-axis is the measured count using the Horiba hematology analyzer, while the y-axis is the count obtained using the CytoTracker. The red dots represent the samples that are outside of the normal range for WBC counts (4.5 k/μl—11 k/μl) or granulocyte counts (1.2 k/μl—6.8 k/μl) [44]. Within the dynamic range, data showed a correlation coefficient (r) value of 0.97 with respect to the reference device (Horiba) in terms of total WBC count and 0.98 in terms of granulocyte count. The correlation coefficient (r) values indicated that the CytoTracker counts have a strong correlation with the counts provided by the benchtop device. Furthermore, we evaluated the difference between the values provided by CytoTracker and Horiba. The Bland–Altman plots showing the agreement between the two instruments are presented in Fig 2. The mean difference between CytoTracker and Horiba in WBC count was 0.82 k/μl with 95% limits of agreement from -1.65 to 3.29 k/μl, and the mean difference in granulocyte count was 0.04 k/μl with 95% limits of agreement from -1.64 to 1.71 k/μl. We calculated the mean bias for the total WBC count and granulocyte count. Bias is defined as the difference between CytoTracker and Horiba counts divided by the Horiba count (Eq 1). As shown in Table 2, the mean bias for the total WBC count is 13.9%, and the mean bias for the granulocyte count is 12.2%, both of which are within CLIA acceptable limits for WBCs and granulocytes of 15% mean bias [45].


$$Bias(\%)=\frac{ABS(CytoTracker\;value-Horiba\;value)}{Horiba\;value}\times 100\%$$ (Eq 1)

To evaluate the device-to-device variation of CytoTracker cartridges, we performed tests on three different cartridges on n = 98 different blood samples. We calculated the coefficient of variation (CV) over three measurements. Table 2 shows the mean CV of WBC count and granulocyte count for 98 samples. The variations on both parameters measured by three new cartridges were within 10%, again within CLIA acceptable limits of 15% CV [45].

**Table 2:** Performance of CytoTracker on linearity test.

Parameter	r	Slope (95% CI)	Intercept (95% CI)	Mean CV	Mean Bias
WBC	0.97	1.05 (1.02, 1.08)	0.41 (0.15, 0.65)	6.07%	13.9%
Granulocyte	0.98	1.04 (1.01,1.07)	-0.15 (-0.30, 0.04)	7.23%	12.2%

### Flagging abnormal blood counts

**Fig 3:**
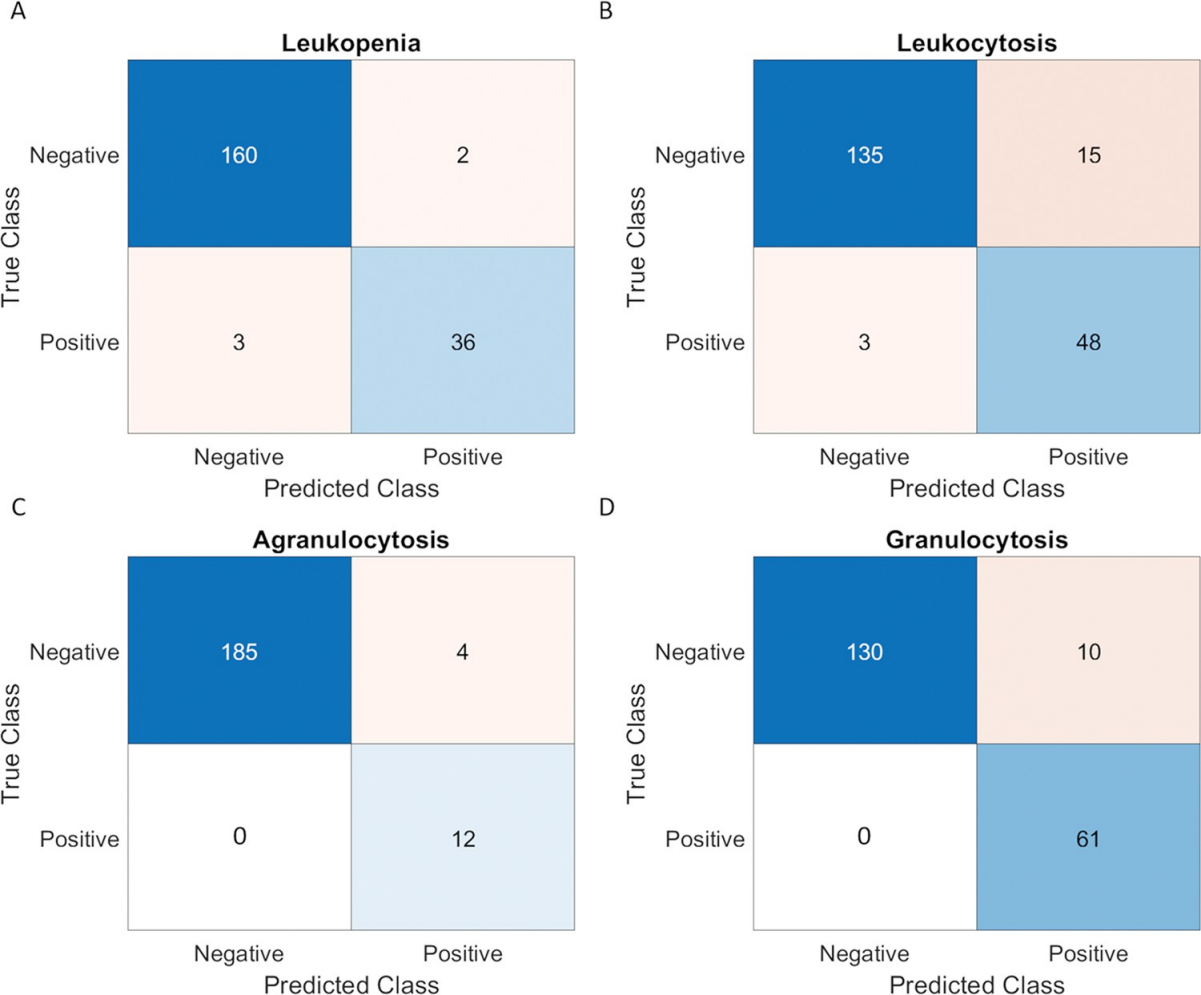
Confusion matrices of CytoTracker for predicting abnormal blood count cases. (A) leukopenia, (B) leukocytosis, (C) agranulocytosis, and (D) granulocytosis. The true class is defined by the counts obtained by Horiba, and the predicted class is defined by the counts obtained by CytoTracker.

We benchmarked CytoTracker accuracy for flagging abnormal white blood counts. The normal range for total WBC counts is 4.5–11 k/μl and for absolute granulocyte counts is 1.2–6.8 k/μl [44]. When choosing the threshold for flagging abnormal cell counts, we factored in the CytoTracker’s device-to-device variation of approximately 10%, and thus set the range for CytoTracker 5% above the lower threshold and 5% below the higher threshold to account for WBC counts that are borderline abnormal, resulting in 4.725–10.45 k/μl for the WBC count range and 1.26–6.46 k/μl for the granulocyte count range. Fig 3 shows the confusion matrices for flagging low WBC counts (leukopenia), high WBC counts (leukocytosis), low granulocyte count (agranulocytosis), and high granulocyte count (granulocytosis). The true counts are defined as those obtained using the comparator device (Horiba), and the predicted counts are those obtained using the CytoTracker. Each confusion matrix was divided into four parts: True positive (TP), False Positive (FP), True Negative (TN), and False Negative (FN). We use sensitivity and specificity to evaluate the CytoTracker’s performance in identifying abnormal blood counts. Sensitivity and specificity along with the percentage of Type I and Type II errors are derived from the TP, TN, and the FP and FN as shown below:

$$Sensitivity=\frac{TP}{TP+FN}$$ (Eq 2)

$$Specificity=\frac{TN}{TN+FP}$$ (Eq 3)

$$Type\;I\;error=\frac{FP}{TN+FP}$$ (Eq 4)

$$Type\;II\;error=\frac{FN}{TP+FN}$$ (Eq 5)

Results are displayed in Table 3. The sensitivity and specificity of all cases are high, and both type I errors and type II errors are low, therefore, demonstrating the CytoTracker’s accuracy in flagging abnormal blood counts.

**Table 3:** Performance of CytoTracker on identifying abnormal counts.

	Sensitivity	Specificity	Type I error	Type II error
Leukopenia	92.3%	98.8%	1.2%	7.7%
Leukocytosis	94.3%	90.0%	10.0%	5.7%
Agranulocytosis	100.0%	97.9%	2.1%	0.0%
Granulocytosis	100.0%	92.9%	7.1%	0.0%

### Blood count difference between inpatient and outpatient

**Fig 4:**
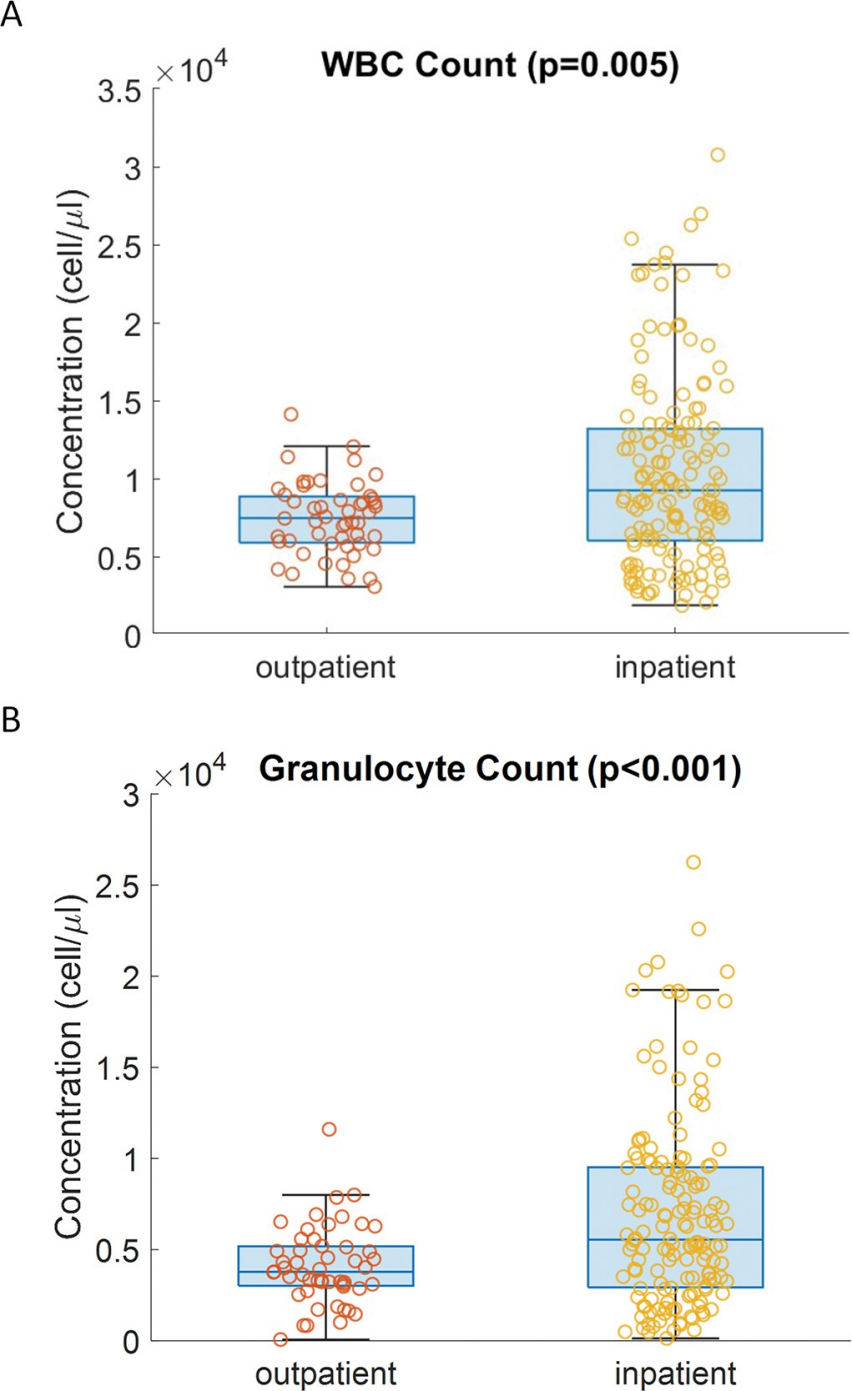
Comparison between inpatients and outpatients based on CytoTracker measurements. (A) total WBC (p = 0.005), (B) granulocyte counts (p<0.001). The circles represent the value of each sample.

We explored whether the CytoTracker has the potential for assessment of infectious disease severity using the device output. We leveraged the fact that our cohort consisted of both outpatients and inpatients. Inpatients tend to present more severe infections than outpatients. In Fig 4, we compare total WBC count and granulocyte count measured by the CytoTracker for both outpatients (mild infection) and inpatients (severe infection). The jittered points in the boxplot represented all the observations for each group (outpatient and inpatient) to visualize the distribution directly. As illustrated in the figure, median total WBC and granulocyte count in inpatient samples are higher than those of outpatient samples. Additionally, for both parameters, the inpatient cohort exhibits more significant variation in comparison to outpatients. The Mann-Whitney U test also showed that the two cohorts exhibit distributions that are distinct from each other with statistical significance (total WBC count p = 0.005; granulocyte count, p<0.001). The same analysis was performed using the counts obtained by the Horiba, also exhibiting statistically significant differences in the in-patient and outpatient cohorts (total WBC count p = 0.016; granulocyte count, p<0.001), confirming the conclusions drawn using the CytoTracker.

## Discussion

Accurate and timely determination of blood count can improve the standard of care for patients. In this work, we conducted a clinical study over three sites to evaluate CytoTracker’s ability to measure the total WBC count and granulocyte count. Samples were collected from 210 patients. The study focused on recruiting outpatients and inpatients with viral and bacterial infections. CytoTracker performance was benchmarked against an FDA-cleared hematology analyzer (Horiba) as the reference device in terms of precision, linearity, mean bias. The Passing-Bablok regression analysis indicates strong agreement between WBC counts obtained from CytoTracker and the comparator device. The correlation coefficient values for the total WBC count and the granulocyte count are 0.97 and 0.98, respectively, thus demonstrating strong linearity. The mean bias of the total WBC count (13.9%) and the granulocyte count (12.2%) are within the Clinical Laboratory Improvement Amendments (CLIA) allowable error (within 15%). Also, we evaluated CytoTracker device-to-device variation measurements by obtaining the mean CV over three cartridges. The variations on both parameters over three cartridges were within 10%, lower than the CLIA’s precision acceptance criteria (15%). The evaluation of the CytoTracker for these three critical parameters validates its ability for accurate and reliable analysis of the total WBC and granulocyte counts.

Additionally, we demonstrate that the CytoTracker can accurately flag abnormal cell counts. We used the value provided by Horiba to determine the true classes. The results reveal that both the sensitivity and specificity of CytoTracker are high for all cases, with low type I and type II errors, and thus low false negative and false positive flagging. One of the limitations of this study was that there was a relatively small sample size of patients with agranulocytosis (essentially neutropenia) and thus patients with borderline low levels of granulocytes (predominantly neutrophils) could get misclassified as normal levels. Future studies will benefit from enrolling a larger sample size of patients with neutropenia. Furthermore, we also assessed differences in cell counts between inpatients and outpatients, demonstrating proof of concept for assessment of infectious disease severity. Inpatients on average show higher mean WBC and granulocyte counts with statistical significance (p < 0.001). With a larger study population, further optimization and assessment can be done using machine learning classification and regression algorithms, so that we will be able to assess infectious disease severity and also differentiate between viral and bacterial infections.

One of the most valuable features of the CytoTracker is the tiny amount of blood required to perform a test (50 μl is sufficient). As mentioned, given the need to ship samples from the clinical sites to RizLab Health for analysis, this study was focused on benchmarking test strip performance using venous blood given the time sensitive stability of capillary blood (limited to a few hours after collection). Future studies will be dedicated to collecting both capillary and venous blood from patients and performing on-site analysis to establish accuracy in analysis of capillary blood and relevant matrix comparison studies.

In conclusion, we developed and clinically validated a miniaturized, portable, and affordable analyzer that can rapidly and accurately measure WBC/granulocyte counts and can greatly improve the standard of care for numerous indications. Future studies will be dedicated to on-site analysis of the device over a larger cohort of patients and clinical validation of additional parameters such as lymphocyte counts. Additional data analytics capabilities, such as infectious disease severity assessment and viral/bacterial differentiation can also be integrated for further benefit to both clinicians and patients.

## Supporting information

S1 Checklist STROBE statement—checklist of items that should be included in reports of observational studies. (DOCX)

S1 Table Clinical study data. (DOCX)
