# Supplementary material for: Clinical evaluation of a fully electronic microfluidic white blood cell analyzer
Source: PLoS One. 2024 Jan 18;19(1):e0296344. doi: 10.1371/journal.pone.0296344 (PMC10796056; doi:10.1371/journal.pone.0296344)
Supplement: S1 Checklist — (DOCX) [file pone.0296344.s001.docx]

STROBE Statement—checklist of items that should be included in reports of observational studies

|  | Item No. | Recommendation | Page  No. | Relevant text from manuscript |
| --- | --- | --- | --- | --- |
| **Title and abstract** | 1 | (*a*) Indicate the study’s design with a commonly used term in the title or the abstract | 1-2 | Clinical evaluation of a fully electronic microfluidic white blood cell analyzer |
|  |  | (*b*) Provide in the abstract an informative and balanced summary of what was done and what was found | 2 | Here, we present the CytoTracker, a fully electronic, microfluidic based instant WBC analyzer with the potential to be used at point-of-care. The CytoTracker is a lightweight, portable, affordable platform capable of quantifying WBCs within minutes using only 50 μl of blood (approximately one drop of blood). In this study, we clinically evaluated the accuracy and performance of CytoTracker in measuring WBC and granulocyte counts. A total of 210 adult patients were recruited in the study. We validated the CytoTracker against a standard benchtop analyzer (Horiba Point of Care Hematology Analyzer, ABX Micros 60). Linear dynamic ranges of 2.5 k/µl – 35 k/µl and 0.6 k/µl – 26 k/µl were achieved for total WBC count and granulocyte count with correlation coefficients of 0.97 and 0.98. In addition, we verified CytoTracker’s capability of identifying abnormal blood counts with above 90% sensitivity and specificity. The promising results of this clinical validation study demonstrate the potential for use of the CytoTracker as a reliable and accurate point-of-care WBC analyzer. |
| Introduction | | | |  |
| Background/rationale | 2 | Explain the scientific background and rationale for the investigation being reported | 3-5 | See the manuscript |
| Objectives | 3 | State specific objectives, including any prespecified hypotheses | 6 | We evaluated the reliability and accuracy of the CytoTracker in measuring WBC and granulocyte counts in patients with confirmed viral and bacterial infections; and investigated the CytoTracker’s accuracy in flagging abnormal blood counts. Additionally, we assessed the differences in cell counts between inpatients and outpatients. |
| Methods | | | |  |
| Study design | 4 | Present key elements of study design early in the paper | 7-8 | See the mauscript |
| Setting | 5 | Describe the setting, locations, and relevant dates, including periods of recruitment, exposure, follow-up, and data collection | 7-8 | We obtained whole venous blood samples from patients from three different clinical sites: Baylor College of Medicine (n=53), Robert Wood Johnson Medical School (n=99) and BioIVT (n=58). BioIVT is a biological product provider. The samples obtained by BioIVT, inc. were from outpatients from a network of clinical sites. The samples obtained from BCM were from emergency room patients (recruited under an IRB for an observational study). The samples obtained from Rutgers RWJ were from emergency room and ICU patients (discarded samples obtained under a retrospective study). Venous blood samples were collected from May 2021 to November 2021. |
| Participants | 6 | (*a*) *Cohort study*—Give the eligibility criteria, and the sources and methods of selection of participants. Describe methods of follow-up  *Case-control study*—Give the eligibility criteria, and the sources and methods of case ascertainment and control selection. Give the rationale for the choice of cases and controls  *Cross-sectional study*—Give the eligibility criteria, and the sources and methods of selection of participants | 8 | All patients enrolled in the study were adults (>18 years) with suspected/confirmed viral (COVID-positive or other viral infection) and bacterial infections (lower UTI, pneumonia, septicemia, appendicitis, and other relevant bacterial infections), confirmed per standard of care (PCR test for viral, culture test for bacterial, or clinical presentation). Excluded from the study were patients with known white blood cell, neutrophil, and lymphocyte disorders, also active cancer patients and patients who received chemotherapy for solid tumors in the last three months, and pregnant patients.  Written consent was obtained by patients recruited by Baylor and BioIVT. The study at Rutgers was retrospective, and thus patient consent was not required. |
|  |  | (*b*) *Cohort study*—For matched studies, give matching criteria and number of exposed and unexposed  *Case-control study*—For matched studies, give matching criteria and the number of controls per case | N/A |  |
| Variables | 7 | Clearly define all outcomes, exposures, predictors, potential confounders, and effect modifiers. Give diagnostic criteria, if applicable | N/A |  |
| Data sources/ measurement | 8* | For each variable of interest, give sources of data and details of methods of assessment (measurement). Describe comparability of assessment methods if there is more than one group | 8 | Venous blood samples were collected from patients for analysis using both the CytoTracker and a Horiba ABX Micros 60 Hematology Analyzer (FDA-cleared device). The sample was stored at 4°C before overnight shipping. The sample tubes were wrapped with an absorbent pad and bubble wrap and placed in a Styrofoam box with cold packs during the shipment. All samples were analyzed at RizLab health within 72 hours after collection from patients. Deidentified information was abstracted from the medical chart including CBC values (assessed by the clinical laboratory), diagnostic lab tests, body temperature, date of sample collection, drug treatments (duration, dose), diagnosis for admission, and clinical status. |
| Bias | 9 | Describe any efforts to address potential sources of bias | N/A |  |
| Study size | 10 | Explain how the study size was arrived at | 8 | A sample size of greater than 150 patients was determined to achieve a high statistical power of greater than 99%. This sample size was selected to detect a significant correlation between the measurements obtained from the two devices. With this large sample size, the study's ability to provide robust evidence regarding the relationship between cell counts is enhanced, supporting the statistical generalizability of the study. |

Continued on next page

| Quantitative variables | 11 | Explain how quantitative variables were handled in the analyses. If applicable, describe which groupings were chosen and why | 9, 11-15 | See the manucript |
| --- | --- | --- | --- | --- |
| Statistical methods | 12 | (*a*) Describe all statistical methods, including those used to control for confounding | 9 | A linear regression was performed to model the correlation between the results acquired by the Horiba ABX micros 60 hematology analyzer and the CytoTracker, and the correlation coefficient was calculated. The level of significance for all statistical tests was a 2-sided, p-value of 0.05. Student’s t-tests were performed to evaluate the difference in total WBC concentration and granulocyte concentration between inpatient blood samples and outpatient blood samples. |
|  |  | (*b*) Describe any methods used to examine subgroups and interactions | N/A |  |
|  |  | (*c*) Explain how missing data were addressed | N/A |  |
|  |  | (*d*) *Cohort study*—If applicable, explain how loss to follow-up was addressed  *Case-control study*—If applicable, explain how matching of cases and controls was addressed  *Cross-sectional study*—If applicable, describe analytical methods taking account of sampling strategy | N/A |  |
|  |  | (*e*) Describe any sensitivity analyses | N/A |  |
| Results | | | | |
| Participants | 13* | (a) Report numbers of individuals at each stage of study—eg numbers potentially eligible, examined for eligibility, confirmed eligible, included in the study, completing follow-up, and analysed | 9 | Samples were obtained from 210 patients with bacterial or viral infections (Fig 1). Nine patients were excluded due to the sample being improperly handled during shipping. CytoTracker performance benchmarking was performed on 201 patient samples. |
|  |  | (b) Give reasons for non-participation at each stage | 9 | Nine patients were excluded due to the sample being improperly handled during shipping. |
|  |  | (c) Consider use of a flow diagram | Fig 1 |  |
| Descriptive data | 14* | (a) Give characteristics of study participants (eg demographic, clinical, social) and information on exposures and potential confounders | 9-10 | Samples were obtained from 210 patients with bacterial or viral infections (Fig 1). Nine patients were excluded due to the sample being improperly handled during shipping. CytoTracker performance benchmarking was performed on 201 patient samples, 51 from BioIVT, 53 from Baylor College of Medicine, and 97 from Robert Wood Johnson Medical School (Table 1). The baseline characteristics of all patients from the three sites are summarized in Table 1. The median age was 54 (Q1-Q3 40-68), with 106 males (53.5%) and 82 females (46.5%). Of the total, 44.4% were white, 17.7% were African American, and 37.9% were patients of other races. |
|  |  | (b) Indicate number of participants with missing data for each variable of interest | N/A |  |
|  |  | (c) *Cohort study*—Summarise follow-up time (eg, average and total amount) | N/A |  |
| Outcome data | 15* | *Cohort study*—Report numbers of outcome events or summary measures over time | N/A |  |
|  |  | *Case-control study—*Report numbers in each exposure category, or summary measures of exposure | N/A |  |
|  |  | *Cross-sectional study—*Report numbers of outcome events or summary measures | N/A |  |
| Main results | 16 | (*a*) Give unadjusted estimates and, if applicable, confounder-adjusted estimates and their precision (eg, 95% confidence interval). Make clear which confounders were adjusted for and why they were included | N/A |  |
|  |  | (*b*) Report category boundaries when continuous variables were categorized | 13 | The normal range for total WBC counts is 4.5 - 11 k/μl and for absolute granulocyte counts is 1.2-6.8 k/μl. When choosing the threshold for flagging abnormal cell counts, we factored in the CytoTracker’s device-to-device variation of approximately 10%, and thus set the range for CytoTracker 5% above the lower threshold and 5% below the higher threshold to account for WBC counts that are borderline abnormal, resulting in 4.725 - 10.45 k/μl for the WBC count range and 1.26 - 6.46 k/μl for the granulocyte count range. |
|  |  | (*c*) If relevant, consider translating estimates of relative risk into absolute risk for a meaningful time period | N/A |  |

Continued on next page

| Other analyses | 17 | Report other analyses done—eg analyses of subgroups and interactions, and sensitivity analyses | N/A |  |
| --- | --- | --- | --- | --- |
| Discussion | | | | |
| Key results | 18 | Summarise key results with reference to study objectives | 16-17 | The correlation coefficient values for the total WBC count and the granulocyte count are 0.97 and 0.98, respectively, thus demonstrating strong linearity. The mean bias of the total WBC count (13.9%) and the granulocyte count (12.2%) are within the Clinical Laboratory Improvement Amendments (CLIA) allowable error (within 15%). Also, we evaluated CytoTracker device-to-device variation measurements by obtaining the mean CV over three cartridges. The variations on both parameters over three cartridges were within 10%, lower than the CLIA's precision acceptance criteria (15%). We used the value provided by Horiba to determine the true classes. The results reveal that both the sensitivity and specificity of CytoTracker are high for all cases, with low type I and type II errors, and thus low false negative and false positive flagging. |
| Limitations | 19 | Discuss limitations of the study, taking into account sources of potential bias or imprecision. Discuss both direction and magnitude of any potential bias | 17-18 | One of the limitations of this study was that there was a relatively small sample size of patients with agranulocytosis (essentially neutropenia) and thus patients with borderline low levels of granulocytes (predominantly neutrophils) could get misclassified as normal levels. Future studies will benefit from enrolling a larger sample size of patients with neutropenia. As mentioned, given the need to ship samples from the clinical sites to RizLab Health for analysis, this study was focused on benchmarking test strip performance using venous blood given the time sensitive stability of capillary blood (limited to a few hours after collection). Future studies will be dedicated to collecting both capillary and venous blood from patients and performing on-site analysis to establish accuracy in analysis of capillary blood and relevant matrix comparison studies. Inpatients on average show higher mean WBC and granulocyte counts with statistical significance (p < 0.001). |
| Interpretation | 20 | Give a cautious overall interpretation of results considering objectives, limitations, multiplicity of analyses, results from similar studies, and other relevant evidence | 16-18 | See the mauscript |
| Generalisability | 21 | Discuss the generalisability (external validity) of the study results | N/A |  |
| Other information | |  | | |
| Funding | 22 | Give the source of funding and the role of the funders for the present study and, if applicable, for the original study on which the present article is based | Provided in the submission system |  |

*Give information separately for cases and controls in case-control studies and, if applicable, for exposed and unexposed groups in cohort and cross-sectional studies.

**Note:** An Explanation and Elaboration article discusses each checklist item and gives methodological background and published examples of transparent reporting. The STROBE checklist is best used in conjunction with this article (freely available on the Web sites of PLoS Medicine at http://www.plosmedicine.org/, Annals of Internal Medicine at http://www.annals.org/, and Epidemiology at http://www.epidem.com/). Information on the STROBE Initiative is available at www.strobe-statement.org.
