# Supplementary material for: Clinical evaluation of a fully electronic microfluidic white blood cell analyzer
Source: PLoS One. 2024 Jan 18;19(1):e0296344. doi: 10.1371/journal.pone.0296344 (PMC10796056; doi:10.1371/journal.pone.0296344)
Supplement: S1 Table — (DOCX) [file pone.0296344.s002.docx]

Supporting Information S1- Clinical study data

**S1 Table.** **Clinical study data**

| # | CytoTracker WBC count (k/μl) | Horiba WBC count (k/μl) | CytoTracker granulocyte count (k/μl) | Horiba granulocyte count (k/μl) |  |
| --- | --- | --- | --- | --- | --- |
| 1 | 8.96 | 8.73 | 6.81 | 6.97 | Outpatient |
| 2 | 2.88 | 2.7 | 1.64 | 1.93 | Outpatient |
| 3 | 9.58 | 9 | 6.92 | 6.57 | Outpatient |
| 4 | 6.2 | 5.8 | 3.1 | 3 | Outpatient |
| 5 | 7.8 | 7.13 | 4.92 | 4.5 | Outpatient |
| 6 | 5.72 | 5.2 | 3.23 | 2.93 | Outpatient |
| 7 | 11.45 | 9.53 | 6.53 | 6.1 | Outpatient |
| 8 | 4.91 | 4.27 | 3.1 | 3.03 | Outpatient |
| 9 | 4.41 | 3.77 | 1.87 | 1.7 | Outpatient |
| 10 | 7.1 | 6.3 | 4.38 | 3.93 | Outpatient |
| 11 | 5.92 | 5.37 | 3.3 | 3.47 | Outpatient |
| 12 | 6.38 | 5.5 | 4.31 | 3.07 | Outpatient |
| 13 | 3.41 | 3.37 | 0.84 | 1.07 | Outpatient |
| 14 | 7.21 | 5.8 | 4.48 | 3.83 | Outpatient |
| 15 | 8.19 | 7 | 6.29 | 5.47 | Outpatient |
| 16 | 10.3 | 8.53 | 6.11 | 4.67 | Outpatient |
| 17 | 8.61 | 8.47 | 3.23 | 3.8 | Outpatient |
| 18 | 8.43 | 6.3 | 4 | 2.83 | Outpatient |
| 19 | 14.27 | 13.37 | 11.6 | 10.73 | Outpatient |
| 20 | 8.87 | 7.43 | 5.58 | 4.77 | Outpatient |
| 21 | 7.39 | 5.87 | 4.95 | 4.57 | Outpatient |
| 22 | 8.33 | 7.57 | 4.29 | 3.8 | Outpatient |
| 23 | 4.31 | 4 | 2.53 | 2.67 | Outpatient |
| 24 | 8.68 | 8.5 | 4.02 | 3.8 | Outpatient |
| 25 | 8.07 | 8.23 | 3.78 | 3.63 | Outpatient |
| 26 | 9.63 | 8.53 | 5.57 | 5.4 | Outpatient |
| 27 | 8.14 | 7.97 | 5.19 | 5.33 | Outpatient |
| 28 | 12.14 | 11.6 | 8 | 7.33 | Outpatient |
| 29 | 3.72 | 3.43 | 1.69 | 1.87 | Outpatient |
| 30 | 5.05 | 4.6 | 2.74 | 2.83 | Outpatient |
| 31 | 5.55 | 5.3 | 2.99 | 2.87 | Outpatient |
| 32 | 9.8 | 9.53 | 6.42 | 6.4 | Outpatient |
| 33 | 20.14 | 19.13 | 16.08 | 14.93 | Inpatient |
| 34 | 6.4 | 6.17 | 2.96 | 2.5 | Inpatient |
| 35 | 5.04 | 3.33 | 1.9 | 1.87 | Inpatient |
| 36 | 11.22 | 7.73 | 5.82 | 5.4 | Inpatient |
| 37 | 3.12 | 2.63 | 1.3 | 1.5 | Inpatient |
| 38 | 3.43 | 2.9 | 1.7 | 1.9 | Inpatient |
| 39 | 7.44 | 6.17 | 5.33 | 4.47 | Inpatient |
| 40 | 9.9 | 6.8 | 3.21 | 3.33 | Outpatient |
| 41 | 9.82 | 8.6 | 5.14 | 5.17 | Outpatient |
| 42 | 4.58 | 4.4 | 0.81 | 1.17 | Outpatient |
| 43 | 11.13 | 9.13 | 7.16 | 5.9 | Inpatient |
| 44 | 3.3 | 2.87 | 1.72 | 1.8 | Inpatient |
| 45 | 8.36 | 7.07 | 6.26 | 5.6 | Inpatient |
| 46 | 6.33 | 5.5 | 3.51 | 3.1 | Outpatient |
| 47 | 5.73 | 5.6 | 2.87 | 3.23 | Outpatient |
| 48 | 3.67 | 3.67 | 0.7 | 1.27 | Inpatient |
| 49 | 3.69 | 3.43 | 2.13 | 2.6 | Inpatient |
| 50 | 6.2 | 5.7 | 3.62 | 2.8 | Outpatient |
| 51 | 9.35 | 8.63 | 6.38 | 6.67 | Outpatient |
| 52 | 6.9 | 5.37 | 3.92 | 3.77 | Outpatient |
| 53 | 8.44 | 7.33 | 3.32 | 3.57 | Outpatient |
| 54 | 6.11 | 5.33 | 1.71 | 1.6 | Outpatient |
| 55 | 7.02 | 6.37 | 4.56 | 4.1 | Outpatient |
| 56 | 8.51 | 7.43 | 4.89 | 3.77 | Outpatient |
| 57 | 7.49 | 7.1 | 3.76 | 4.13 | Outpatient |
| 58 | 3.12 | 2.27 | 0.91 | 1.13 | Inpatient |
| 59 | 14.66 | 12.67 | 10.52 | 8.73 | Inpatient |
| 60 | 10.39 | 7.93 | 7.45 | 6.3 | Inpatient |
| 61 | 3.26 | 2.33 | 1.42 | 1.2 | Inpatient |
| 62 | 9.53 | 7.83 | 5.06 | 4.83 | Inpatient |
| 63 | 2.57 | 1.6 | 0.78 | 0.87 | Inpatient |
| 64 | 23.49 | 22.17 | 19.24 | 19.87 | Inpatient |
| 65 | 13.64 | 11.6 | 9.05 | 8.6 | Inpatient |
| 66 | 8.01 | 6.57 | 5.26 | 4.73 | Inpatient |
| 67 | 2.41 | 1.73 | 0.88 | 0.87 | Inpatient |
| 68 | 12.98 | 11.7 | 9.4 | 9.7 | Inpatient |
| 69 | 7.13 | 6.27 | 1.01 | 0.93 | Outpatient |
| 70 | 6.2 | 5.13 | 2.54 | 2.53 | Inpatient |
| 71 | 7.75 | 7.47 | 5.13 | 5.63 | Inpatient |
| 72 | 16.47 | 11.3 | 10.07 | 8.53 | Inpatient |
| 73 | 2.32 | 2 | 1.32 | 1.6 | Inpatient |
| 74 | 15.4 | 14.6 | 11.31 | 10.2 | Inpatient |
| 75 | 26.78 | 25.6 | 22.59 | 22.13 | Inpatient |
| 76 | 13.72 | 12.07 | 9.54 | 8.7 | Inpatient |
| 77 | 27.52 | 28.27 | 20.26 | 22.37 | Inpatient |
| 78 | 10 | 8.03 | 7.49 | 6.43 | Inpatient |
| 79 | 6.4 | 5.67 | 5.51 | 3.67 | Inpatient |
| 80 | 5.98 | 5.53 | 3.94 | 4.4 | Inpatient |
| 81 | 7.73 | 6.47 | 5.01 | 4.57 | Inpatient |
| 82 | 16.01 | 14.83 | 6.83 | 8.07 | Inpatient |
| 83 | 9.26 | 9.1 | 5.58 | 6.17 | Inpatient |
| 84 | 12.96 | 11.57 | 9.8 | 9.57 | Inpatient |
| 85 | 16.13 | 15 | 12.96 | 13.43 | Inpatient |
| 86 | 3.93 | 3.77 | 2.46 | 2.97 | Inpatient |
| 87 | 10.03 | 15.47 | 6.55 | 8.5 | Inpatient |
| 88 | 5.92 | 4.87 | 3.49 | 3.47 | Inpatient |
| 89 | 23.77 | 23.47 | 18.64 | 19.53 | Inpatient |
| 90 | 8.52 | 4.63 | 5.98 | 2.97 | Inpatient |
| 91 | 4.51 | 5.4 | 2.39 | 2.9 | Inpatient |
| 92 | 18.83 | 18.7 | 13.2 | 12.67 | Inpatient |
| 93 | 13.35 | 12.17 | 8.17 | 6.93 | Inpatient |
| 94 | 25.88 | 24.7 | 19.19 | 18.27 | Inpatient |
| 95 | 19.91 | 20.73 | 14.38 | 14.73 | Inpatient |
| 96 | 20.08 | 17.37 | 15.41 | 14.23 | Inpatient |
| 97 | 1.85 | 1.13 | 0.57 | 0.6 | Inpatient |
| 98 | 11.8 | 10.83 | 6.9 | 6.6 | Inpatient |
| 99 | 31.43 | 31.3 | 26.25 | 26.5 | Inpatient |
| 100 | 18.09 | 16.93 | 14.34 | 15.2 | Inpatient |
| 101 | 11.25 | 13.73 | 7.87 | 7.67 | Outpatient |
| 102 | 5.87 | 7.5 | 0.07 | 1.17 | Outpatient |
| 103 | 3.62 | 2.2 | 0.59 | 0.7 | Inpatient |
| 104 | 8.6 | 7.43 | 5.24 | 5.33 | Inpatient |
| 105 | 12.33 | 11.1 | 7.31 | 6.4 | Inpatient |
| 106 | 4.21 | 2.97 | 1.86 | 1.97 | Inpatient |
| 107 | 9.84 | 8.13 | 5.41 | 5.3 | Inpatient |
| 108 | 12.42 | 11.27 | 8.23 | 7.93 | Inpatient |
| 109 | 19.17 | 15.37 | 15.01 | 12.53 | Inpatient |
| 110 | 7.97 | 8.1 | 4.44 | 4.87 | Inpatient |
| 111 | 9.22 | 8.77 | 6.38 | 6.23 | Inpatient |
| 112 | 15.59 | 20.8 | 10.56 | 14.23 | Inpatient |
| 113 | 12.45 | 11.6 | 9.5 | 9.27 | Inpatient |
| 114 | 2.84 | 2.53 | 1.57 | 2.03 | Inpatient |
| 115 | 2.55 | 1.93 | 0.48 | 0.83 | Inpatient |
| 116 | 12.55 | 10.13 | 8.42 | 6.37 | Inpatient |
| 117 | 11.08 | 11.17 | 8.46 | 8.8 | Inpatient |
| 118 | 5.37 | 4.97 | 3.34 | 3.8 | Outpatient |
| 119 | 12.79 | 10.87 | 9.93 | 9.27 | Inpatient |
| 120 | 8.6 | 6.73 | 5.61 | 4.93 | Inpatient |
| 121 | 9.18 | 7.55 | 4.47 | 4.1 | Inpatient |
| 122 | 13.09 | 10.25 | 8.69 | 8.5 | Inpatient |
| 123 | 7.42 | 7.1 | 3.14 | 3.2 | Inpatient |
| 124 | 8.28 | 7.3 | 5.17 | 5.1 | Inpatient |
| 125 | 3.4 | 3.1 | 1.49 | 1.9 | Inpatient |
| 126 | 10.43 | 13.7 | 7.3 | 8.7 | Inpatient |
| 127 | 12.85 | 10.2 | 8.63 | 8.1 | Inpatient |
| 128 | 10.08 | 8.5 | 4.44 | 4.2 | Inpatient |
| 129 | 4.03 | 3.7 | 1.46 | 1.7 | Outpatient |
| 130 | 7.87 | 6.3 | 3.23 | 3.5 | Outpatient |
| 131 | 2.41 | 1.9 | 0.49 | 1.1 | Inpatient |
| 132 | 4.27 | 4 | 2.1 | 2.6 | Inpatient |
| 133 | 5.06 | 4.7 | 2.28 | 2.05 | Inpatient |
| 134 | 3.78 | 3 | 1.89 | 2.1 | Inpatient |
| 135 | 7.57 | 7.2 | 3.76 | 4.3 | Inpatient |
| 136 | 6.88 | 6.1 | 4.98 | 5.2 | Inpatient |
| 137 | 4.61 | 3.7 | 2.9 | 2.8 | Inpatient |
| 138 | 2.59 | 2.1 | 1.23 | 1.3 | Inpatient |
| 139 | 7.5 | 7.4 | 5.3 | 6.3 | Inpatient |
| 140 | 9.52 | 7.6 | 6.32 | 5.6 | Inpatient |
| 141 | 8.16 | 8 | 5.04 | 5.4 | Inpatient |
| 142 | 22.88 | 22.1 | 18.59 | 18.8 | Inpatient |
| 143 | 5.65 | 5 | 2.83 | 2.7 | Inpatient |
| 144 | 16.37 | 15.4 | 9.57 | 8.7 | Inpatient |
| 145 | 2.93 | 1.8 | 1.08 | 1.3 | Inpatient |
| 146 | 5.89 | 5.8 | 3.46 | 3.9 | Inpatient |
| 147 | 1.63 | 1.4 | 0.13 | 0.6 | Inpatient |
| 148 | 4.4 | 3.5 | 1.71 | 2 | Inpatient |
| 149 | 11.05 | 9.9 | 8.59 | 8.3 | Inpatient |
| 150 | 9.25 | 8.9 | 6.28 | 6.5 | Inpatient |
| 151 | 24.3 | 23.3 | 20.32 | 20.9 | Inpatient |
| 152 | 8.16 | 7.9 | 4.78 | 5.7 | Inpatient |
| 153 | 3.83 | 3.2 | 2.6 | 2.5 | Inpatient |
| 154 | 8.02 | 8 | 3.84 | 4.7 | Inpatient |
| 155 | 11.94 | 10.5 | 7.05 | 7.1 | Inpatient |
| 156 | 23.48 | 21.1 | 19.15 | 19.3 | Inpatient |
| 157 | 12.83 | 13 | 9.65 | 10.1 | Inpatient |
| 158 | 6.62 | 6.8 | 4.45 | 5.3 | Inpatient |
| 159 | 5.37 | 4.8 | 2.87 | 3.2 | Inpatient |
| 160 | 6.01 | 5.2 | 4.37 | 4.2 | Inpatient |
| 161 | 11.98 | 10 | 7.12 | 7.1 | Inpatient |
| 162 | 8.78 | 7.7 | 6.41 | 5.3 | Inpatient |
| 163 | 14.13 | 15.2 | 9.28 | 10.9 | Inpatient |
| 164 | 6.79 | 6 | 5.18 | 5.4 | Inpatient |
| 165 | 10.3 | 8.9 | 7.76 | 7.6 | Inpatient |
| 166 | 14.67 | 12.35 | 11.05 | 10.75 | Inpatient |
| 167 | 3.51 | 2.4 | 1.55 | 1.5 | Inpatient |
| 168 | 5.38 | 4.15 | 3.43 | 3.45 | Inpatient |
| 169 | 10 | 9 | 7.26 | 7.55 | Inpatient |
| 170 | 4.29 | 3.65 | 0.97 | 1.2 | Inpatient |
| 171 | 6.8 | 5.65 | 3.4 | 3 | Inpatient |
| 172 | 13.22 | 11.8 | 9.8 | 9.6 | Inpatient |
| 173 | 11.99 | 10.4 | 9.48 | 8.9 | Inpatient |
| 174 | 19.23 | 18.37 | 15.62 | 16.87 | Inpatient |
| 175 | 4.58 | 4.6 | 1.79 | 2.6 | Inpatient |
| 176 | 20.2 | 20.2 | 13.64 | 14.25 | Inpatient |
| 177 | 6.01 | 5.2 | 3.39 | 3.6 | Inpatient |
| 178 | 8.34 | 11.65 | 2.83 | 6.35 | Inpatient |
| 179 | 23.59 | 22.2 | 16.15 | 17.95 | Inpatient |
| 180 | 6.92 | 7.4 | 3.85 | 4.23 | Inpatient |
| 181 | 8.49 | 8.45 | 4.24 | 4.2 | Inpatient |
| 182 | 11.48 | 9.95 | 8.95 | 9 | Inpatient |
| 183 | 11.07 | 10.55 | 7.07 | 6.65 | Inpatient |
| 184 | 10.21 | 9.2 | 5.29 | 5.4 | Inpatient |
| 185 | 13.64 | 11 | 10.28 | 9.27 | Inpatient |
| 186 | 13.7 | 12.9 | 10.01 | 10.15 | Inpatient |
| 187 | 8.24 | 7.8 | 2.6 | 3.3 | Inpatient |
| 188 | 3.07 | 2.5 | 1.76 | 1.8 | Inpatient |
| 189 | 17.37 | 18.45 | 11.11 | 11.75 | Inpatient |
| 190 | 16.25 | 15.1 | 12.22 | 12.25 | Inpatient |
| 191 | 14.39 | 13.2 | 10.96 | 9.73 | Inpatient |
| 192 | 13.37 | 11.1 | 7.47 | 7.6 | Inpatient |
| 193 | 10.05 | 7.2 | 4.72 | 4.7 | Inpatient |
| 194 | 8.52 | 5.7 | 6.59 | 4.8 | Inpatient |
| 195 | 24.17 | 20.4 | 18.97 | 17.4 | Inpatient |
| 196 | 7.42 | 6.47 | 5.22 | 5 | Inpatient |
| 197 | 7.59 | 6.95 | 5.38 | 4.15 | Inpatient |
| 198 | 11.93 | 14.05 | 10 | 12.75 | Inpatient |
| 199 | 6.41 | 4.65 | 3.66 | 2.85 | Inpatient |
| 200 | 9.46 | 8.95 | 3.27 | 5.4 | Inpatient |
| 201 | 6.9 | 6.75 | 3.52 | 4.2 | Inpatient |
